# Factors associated with uptake of gynaecological care and cervical cancer screening among women in the Swiss HIV Cohort Study

**DOI:** 10.1111/hiv.70137

**Published:** 2025-11-11

**Authors:** Mailin Waldecker, Katayoun Taghavi, Chloe Pasin, Philip E. Tarr, Anna Hachfeld, Aline Munting, Irene A. Abela, Maja Weisser, Bettina Schlatter, Olivier Nawej, Enos Bernasconi, Eliane Rohner, Karoline Aebi‐Popp, the Swiss HIV Cohort Study (SHCS), IA Abela, K Aebi‐Popp, A Anagnostopoulos, M Battegay, E Bernasconi, DL Braun, HC Bucher, A Calmy, M Cavassini, A Ciuffi, G Dollenmaier, M Egger, L Elzi, JS Fehr, J Fellay, S Frigerio Malossa, H Furrer, CA Fux, HF Günthard, A Hachfeld, DHU Haerry, B Hasse, HH Hirsch, M Hoffmann, I Hösli, M Huber, D Jackson‐Perry, CR Kahlert, O Keiser, T Klimkait, RD Kouyos, H Kovari, K Kusejko, ND Labhardt, K Leuzinger, B Martinez de Tejada, C Marzolini, KJ Metzner, N Müller, J Nemeth, D Nicca, J Notter, P Paioni, G Pantaleo, M Perreau, A Rauch, LP Salazar‐Vizcaya, P Schmid, O Segeral, RF Speck, M Stöckle, PE Tarr, A Trkola, G Wandeler, M Weisser, S Yerly

**Affiliations:** ^1^ Department of Gynecology and Obstetrics University Hospital of Bern and University of Bern Bern Switzerland; ^2^ Institute of Social and Preventive Medicine University of Bern Bern Switzerland; ^3^ Early Detection, Prevention and Infections Branch, International Agency for Research on Cancer World Health Organization (IARC/WHO) Lyon France; ^4^ Institute of Medical Virology University of Zurich Zurich Switzerland; ^5^ SHARE Collaborative, Blizard Institute Queen Mary University of London London UK; ^6^ University Center for Internal Medicine and Infectious Diseases Service, Kantonsspital Baselland University of Basel Basel Switzerland; ^7^ Department of Infectious Diseases, University Hospital Bern University of Bern Bern Switzerland; ^8^ Service of Infectious Diseases Lausanne University Hospital and University of Lausanne Lausanne Switzerland; ^9^ Department of Infectious Diseases and Hospital Epidemiology University Hospital Zurich Zurich Switzerland; ^10^ Division of Infectious Diseases University Hospital Basel Basel Switzerland; ^11^ HIV Unit, Infectious Diseases Division, Department of Medicine University Hospital of Geneva Geneva Switzerland; ^12^ Division of Infectious Diseases, Ente Ospedaliero Cantonale University of Geneva and University of Southern Switzerland Lugano Switzerland; ^13^ Department of Obstetrics and Gynecology Lindenhofspital Bern Switzerland

**Keywords:** cervical cancer, cervical dysplasia, gynaecological screening, HPV, woman living with HIV

## Abstract

**Objectives:**

We assessed factors associated with attendance at gynaecological visits and cervical cancer screening, and estimated the incidence of cervical dysplasia and cancer among women with HIV (WWH) in Switzerland over two decades.

**Methods:**

We used self‐reported gynaecological information, collected biannually, in the Swiss HIV Cohort Study between April 2001 and June 2022. We used mixed‐effects logistic regression to examine factors associated with attending yearly gynaecological visits and having cervical smears performed. We estimated cervical dysplasia and cancer incidence rates per 100 000 person‐years and used Cox regression to assess factors associated with incident dysplasia and cancer.

**Results:**

Among 4052 included WWH, cervical smears were collected in 83% of 33 097 pregnancy‐unrelated visits. Gynaecological visits were less common among older women, among those with lower education, or with a history of intravenous drug use. If a gynaecological visit occurred, cervical smears were less common among women of Black than White ethnicity. Among 3970 women included in the incidence analysis, 218 cervical dysplasias (crude rate: 466/100 000 person‐years) and 14 cervical cancers (crude rate: 28/100 000 person‐years) were recorded. Women who had cervical smears documented in a higher proportion of time periods were more likely to have a cervical dysplasia diagnosis but less likely to have a cervical cancer diagnosis documented.

**Conclusions:**

We found substantial disparities in the uptake of gynaecological visits and cervical smears by age, education level, ethnicity and intravenous drug use. Implementing more targeted and integrated cervical cancer screening and gynaecological care models may help reduce these disparities and improve prevention of cervical cancer among WWH in Switzerland.

## INTRODUCTION

Virtually all cervical cancers are caused by high‐risk genotypes of human papillomavirus (hrHPV) [1], making the disease largely preventable through vaccination and effective screening. Nevertheless, cervical cancer still causes nearly 27 000 deaths annually in Europe [2]. Women with HIV (WWH) are disproportionately affected, with an approximately six‐fold higher risk of developing cervical cancer compared to women without HIV [3]. This elevated risk is partly explained by the higher prevalence and persistence of hrHPV among WWH [4].

Cervical cancer screening guidelines differ across Europe, reflecting wide variations in health care systems and availability of resources [5]. Furthermore, existing guidelines often leave important questions unanswered. For example, a position paper issued by the European Society of Gynaecological Oncology (ESGO) and the European Federation of Colposcopy (EFC) did not specify screening intervals or the optimal age at which to initiate screening [6]. Notably, the position paper also lacked specific recommendations for WWH. Guidance for WWH is available from the European AIDS Clinical Society, which recommends cytology‐based screening every 1–3 years for WWH above the age of 21 years [7]. On the other hand, the World Health Organization (WHO) cervical cancer screening guideline published in 2021 recommends hrHPV testing as the primary screening test for all women including WWH and an additional follow‐up test for WWH who test positive for hrHPV [8]. The guideline proposes that WWH should undergo hrHPV‐based screening every 3–5 years from the age of 25 years [8].

In Switzerland, the age‐standardized cervical cancer incidence rate (4 per 100 000 women) is less than half the average rate observed across Europe (11 per 100 000 women) [2], although cervical cancer screening is opportunistic and no systematic national or regional screening programmes exist. As in other countries, cervical cancer screening methods have evolved over time, from cytology‐based screening to include hrHPV testing. For many years the Swiss Society of Gynaecology and Obstetrics (SSGO) recommended yearly cytology‐based screening from the age of 21 years, but in 2018 the interval was reduced to every 3 years [9]. For women aged 30 years or older, either primary hrHPV testing or cytology‐based screening every 3 years is recommended. Of note, in Switzerland, cytology‐based but not hrHPV screening is covered by health insurance. Although the SSGO acknowledges that women with immunosuppression are at increased risk of cervical dysplasia, no specific screening recommendations for WWH are provided [9]. A study published almost 20 years ago found that gynaecological care including cervical cancer screening among WWH in Switzerland was insufficient and needed to be improved [10]. We used data from the Swiss HIV Cohort Study (SHCS) to examine whether gynaecological care among WWH in Switzerland has improved over time and to identify factors associated with the uptake of gynaecological visits and cervical smears.

## METHODS

### Data source and inclusion criteria

The SHCS was established in 1988 and is an ongoing, prospective, multicentre, nationwide cohort study of people with HIV [11]. Participants are followed up twice a year at one of seven study centres, affiliated regional hospitals, or private practices. Since 2001, participating women are asked to complete a questionnaire about gynaecological examinations at each visit. If no gynaecological examinations were reported, physicians may have reminded the women or assisted them in scheduling a gynaecological visit. However, data on whether such reminders or scheduling assistance were actually provided were not available. Women could choose to undergo gynaecological examinations within the study centres or separate private practices, depending on their preference. Local ethics committees approved the SHCS, and all participants provided written informed consent. Women who completed at least one gynaecological SHCS questionnaire between 1 April 2001 and 30 June 2022, while not pregnant, were included in the analysis. Women with a history of cervical cancer or hysterectomy before the first gynaecological questionnaire were excluded.

### Statistical analysis

We used descriptive statistics to assess sociodemographic and medical characteristics among the included women. To examine the uptake of gynaecological examinations and cervical smears within the SHCS, we divided the entire study period (1 April 2001 until 30 June 2022) into 17 time periods of 15 months each. In accordance with a previous analysis examining cervical cancer screening uptake within the SHCS between 2001 and 2004 [10], we chose 15‐month time intervals because, within this time frame, most women undergo two SHCS follow‐up visits. We used univariable and multivariable mixed‐effects logistic regression, allowing for random effects at the centre and individual level, to assess participant‐level factors associated with (i) uptake of gynaecological examinations and (ii) provision of cervical smears among those who underwent a gynaecological examination. We examined the following participant‐level factors: current age (18–39, 40–49, 50–59, ≥60 years), ethnicity (White, Black, other), education (mandatory school or less, apprenticeship, bachelor or more), a history of intravenous drug use (yes/no), current CD4 cell count (<200, 200–499, ≥500 cells/μL), and calendar period of follow‐up visit (2001–2004, 2005–2009, 2010–2014, 2015–2019, 2020–2022). We defined baseline as the date of their first gynaecological SHCS questionnaire.

We calculated cervical dysplasia and cervical cancer incidence rates by dividing the recorded number of cervical dysplasia and cervical cancer diagnoses by the number of person‐time at risk. Detailed information on the dysplasia grade or the cervical cancer stage at diagnosis was generally not available. Person‐time at risk was measured from SHCS registration or 1 April 2001, whichever occurred later, to cervical dysplasia or cervical cancer diagnosis, transfer out, loss to follow‐up, death, or database closure, whichever occurred first. Women without follow‐up time within the study period (1 April 2001 until 30 June 2022) were excluded from this analysis. We used Cox proportional hazards models to identify risk factors for cervical dysplasia and cervical cancer including current age (18–39, 40–49, 50–59, ≥60 years), ethnicity (White, Black, other), education (mandatory school or less, apprenticeship, bachelor or more), a history of intravenous drug use (yes/no), smoking at baseline (yes/no), current calendar period (2001–2004, 2005–2009, 2010–2014, 2015–2019, 2020–2022), the percentage of 15‐months periods with a cervical smear recorded (≤25%, 26–50%, 51–75%, >75%), antiretroviral therapy (ART) use (yes/no; time‐updated), and the CD4 cell count at SHCS registration (continuous variable). In the analysis of cervical cancer, we also examined the association with a previous cervical dysplasia diagnosis. All analyses were performed using Stata Version 16 (StataCorp, College Station, TX, USA).

## RESULTS

### Study population

Of 4231 WWH aged 18 years or older with follow‐up visits in the SHCS between 1 April 2001 and 30 June 2022, we excluded 92 women who had had a hysterectomy, 73 women because they had no or only pregnancy‐related gynaecological questionnaires filled in and 13 women because of a prior cervical cancer diagnosis. Overall, we included 4052 WWH with at least one completed gynaecological questionnaire between 1 April 2001 and 30 June 2022. The median baseline age was 36 years (interquartile range [IQR] 31–42). The majority of women were of White ethnicity (*n* = 2249; 55%) and had their first questionnaire filled between 2001 and 2004 (*n* = 2149; 53%), see Table 1. About one in five women had a history of intravenous drug use and 40% were active smokers at baseline. The median baseline CD4 cell count was 400 cells/μL (IQR 249–607).

**TABLE 1:** Sociodemographic and medical characteristics of 4052 included women at the time of their first gynaecological SHCS questionnaire (baseline).

Characteristic	*N* (%)
Median age at baseline [years] (IQR)	36 (31–42)
Calendar year at baseline	
2001–2004	2149 (53%)
2005–2009	828 (20%)
2010–2014	594 (15%)
2015–2019	383 (9%)
2020–2022	98 (2%)
Ethnicity	
White	2249 (55%)
Black	1355 (33%)
Other	427 (11%)
Missing	21 (1%)
Education	
Mandatory school or less	1701 (42%)
Apprenticeship	1411 (35%)
Bachelor or more	754 (19%)
Missing	186 (5%)
History of intravenous drug use	
No	3126 (77%)
Yes	897 (22%)
Missing	29 (1%)
Active smoker at baseline^a^	
No	2377 (59%)
Yes	1639 (40%)
Missing	36 (1%)
Casual sexual partners reported at baseline^a^	
No	3600 (89%)
Yes	367 (9%)
Missing	85 (2%)
Median BMI at baseline^a^ (kg/m^2^)	22 (20–26)
Missing	60 (1%)
Median CD4 cell count at baseline^a^ (cells/μL)	400 (249–607)
Missing	9 (<1%)
ART initiation at some point during follow‐up	3870 (96%)

Abbreviations: BMI, body mass index; IQR, interquartile range.

^a^ Reported within 6 months before or after the first gynaecological questionnaire.

### Gynaecological visits and cervical smears

A total of 35 025 gynaecological visits were reported, with a median of 7 visits (IQR 3–13) per woman during a median follow‐up time of 12 years (IQR 5–19). Most of these visits (*n* = 33 097; 94%) were unrelated to pregnancy. During 83% (*n* = 27 576) of the pregnancy‐unrelated visits, cervical smears were obtained; for 8% (*n* = 2,627), it was unclear whether a cervical smear was taken. Abnormal gynaecological findings were recorded for 17% (*n* = 5717) of gynaecological visits.

**FIGURE 1:**
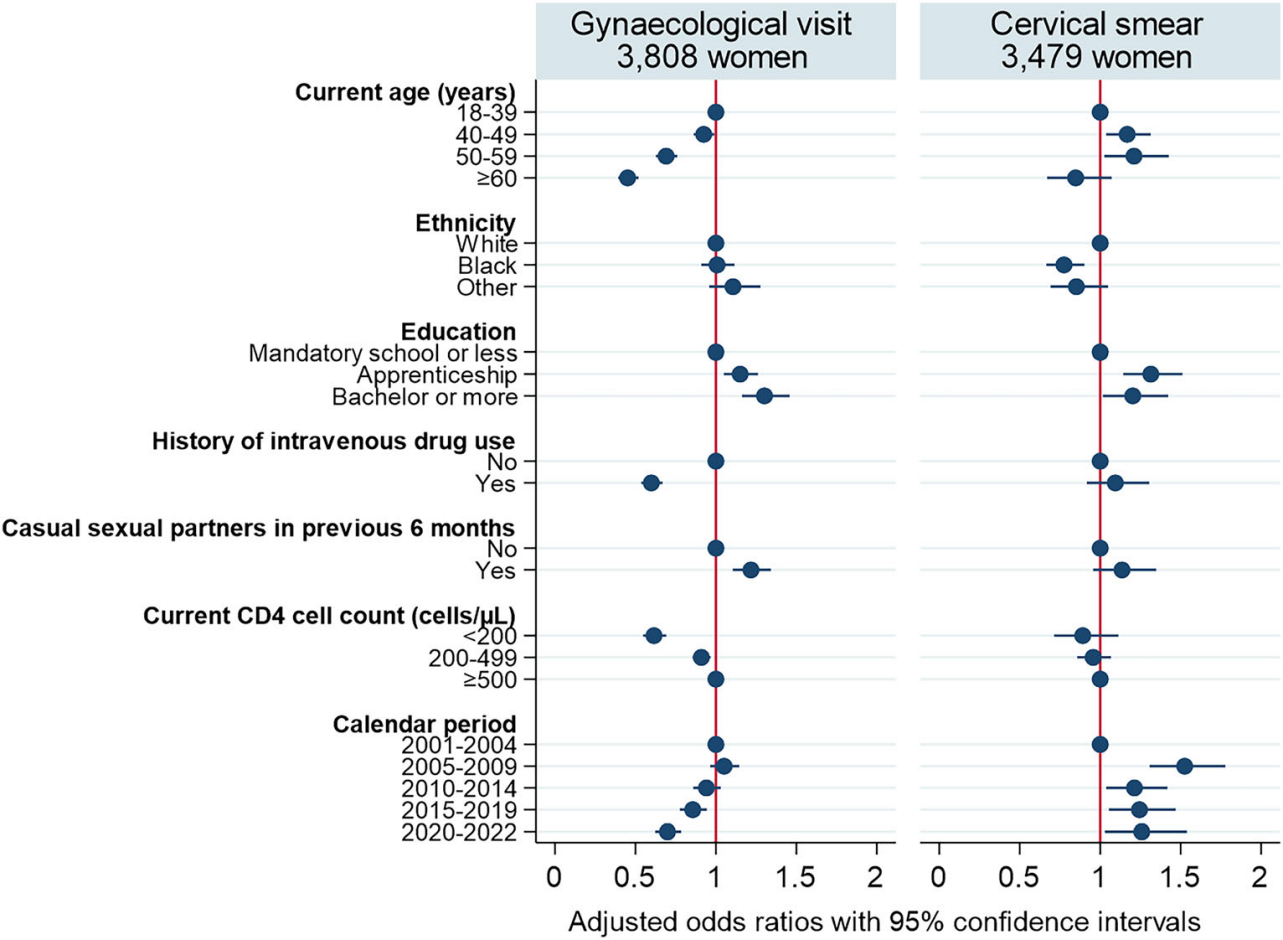
Factors potentially associated with undergoing gynaecological visits or cervical smears (among those with a gynaecological visit) in a given 15‐month period among women with HIV in the SHCS.

The odds of reporting at least one gynaecological visit during a given 15‐month period decreased with older age. That is, compared with women aged 18–39 years, the adjusted odds were 8% lower among those aged 40–49 years (adjusted odds ratio [aOR] 0.92; 95% confidence interval [CI] 0.86–0.99), 31% among those aged 50–59 years (aOR 0.69; 95% CI 0.63–0.76), and 55% lower among women aged 60 years or older (aOR 0.45; 95% CI 0.39–0.52). The likelihood of reporting a gynaecological visit also increased with higher education levels (bachelor or more vs. mandatory school or less; aOR 1.29; 95% CI 1.15–1.45), see Figure 1. Women with a history of intravenous drug use were substantially less likely to undergo a gynaecological visit in a given period than women without a history of intravenous drug use (aOR 0.59, 95% CI 0.53–0.66), whereas casual sexual partners in the previous 6 months were positively associated with gynaecological visits (aOR 1.22; 95% CI 1.11–1.34). Women with low current CD4 cell counts were less likely to report gynaecological visits (<200 vs. ≥500 cells/μL; aOR 0.73; 95% CI 0.66–0.82). The likelihood of reporting gynaecological visits decreased in recent years and was particularly low in the years 2020–2022 compared with 2001–2004 (aOR 0.76; 95% CI 0.68–0.85). In crude analysis, women of Black or other ethnicity were more likely to report gynaecological visits (Table S1), but this association disappeared in the adjusted analysis (Figure 1).

Among women with a gynaecological visit in a given period, middle‐aged women were more likely to undergo a cervical smear than young women (40–49 vs. 18–39 years; aOR 1.16; 95% CI 1.03–1.31), whereas elderly women tended to be less likely to have a cervical smear taken (aOR 0.84; 95% CI 0.66–1.06), see Figure 1. In the crude analysis, Black ethnicity was associated with a slightly increased likelihood of undergoing a cervical smear (crude OR 1.15; 95% CI 1.06–1.26; Table S2), but in the adjusted analysis, women of Black ethnicity were less likely to have had a cervical smear taken than women of White ethnicity (aOR 0.77; 95% CI 0.66–0.89). Higher education was associated with increased odds of reporting a cervical smear (bachelor or more vs. mandatory school or less; aOR 1.19; 95% CI 1.00–1.40). In the adjusted analysis, cervical smears were more common in recent years than in the early calendar period 2001–2004 (Figure 1). Intravenous drug use history, casual sexual partners, or current CD4 cell counts were not clearly associated with reporting a cervical smear in a given period.

### Cervical dysplasia and cervical cancer incidence rates

For the incidence analyses, we excluded 82 women without follow‐up information. Among the remaining 3970 women, 216 were diagnosed with incident cervical dysplasia (crude incidence rate: 462/100 000 person‐years; 95% CI 404–528) and 14 with cervical cancer (crude incidence rate: 28/100 000 person‐years; 95% CI 17–48). The risk of developing cervical dysplasia decreased with older age (≥50 vs. 18–39 years; adjusted hazard ratio [aHR] 0.46; 95% CI 0.26–0.80), and it was higher among women of Black (aHR 1.48; 95% CI 1.04–2.10) and other ethnicities (aHR 1.46; 95% CI 0.95–2.25) than women of White ethnicity (Table 2). Women who were smokers at baseline were more likely to develop cervical dysplasia (aHR 1.77; 95% CI 1.29–2.42), while those with higher CD4 cell counts at registration into the SHCS had a lower cervical dysplasia risk (per 100 cells/μL increase; aHR 0.95; 95% CI 0.90–1.00). There was no clear trend in cervical dysplasia rates over the current calendar period, but between 2020 and 2022 diagnosis rates of cervical dysplasia tended to be lower than in previous years. Women who underwent cervical smears in a higher proportion of time periods were also more likely to have a cervical dysplasia diagnosis recorded (>75% time periods with a smear vs. ≤25%; aHR 8.12; 95% CI 3.91–16.85). In contrast, their risk of developing cervical cancer tended to be lower when the proportion of time periods with a cervical smear increased (Table 2). Cervical cancer rates decreased with increasing CD4 cell counts (per 100 cells/μL increase; aHR 0.77; 95% CI 0.60–0.98).

**TABLE 2:** Crude and adjusted hazard ratios (HR) for developing cervical dysplasia (*N* = 216) and cervical cancer (*N* = 14) among women with HIV in the SHCS.

	Cervical dysplasia		Cervical cancer	
	Crude HR (95% CI)	Adjusted HR (95% CI)	Crude HR (95% CI)	Adjusted HR (95% CI)
	*N* = 3970*	*N* = 3887	*N* = 3970*	*N* = 3887
**Current age (years)**				
18–39	1	1	1	1
40–49	0.71 (0.52–0.97)	0.76 (0.55–1.06)	2.45 (0.68–8.79)	1.90 (0.51–7.01)
≥50	0.34 (0.20–0.59)	0.46 (0.26–0.80)	2.08 (0.44–9.92)	1.14 (0.22–5.90)
**Ethnicity**				
White	1	1	1	1
Black	1.34 (1.00–1.79)	1.48 (1.04–2.10)	0.49 (0.14–1.76)	0.45 (0.11–1.93)
Other	1.52 (1.01–2.30)	1.46 (0.95–2.25)	–	–
**Education**				
Mandatory school or less	1	–	1	–
Apprenticeship	0.88 (0.65–1.19)	–	1.37 (0.42–4.48)	–
Bachelor or more	0.89 (0.61–1.29)	–	0.91 (0.18–4.69)	–
**History of intravenous drug use**				
No	1	–	1	–
Yes	1.01 (0.74–1.40)	–	1.98 (0.66–5.90)	–
**Current smoker at baseline**				
No	1	1	1	1
Yes	1.22 (0.93–1.59)	1.77 (1.29–2.42)	1.44 (0.50–4.10)	0.97 (0.30–3.16)
**Current calendar period**				
2001–2004	1	1	1	–
2005–2009	1.48 (1.02–2.15)	1.44 (0.98–2.11)	1.72 (0.39–7.67)	–
2010–2014	1.29 (0.86–1.95)	1.30 (0.85–2.00)	0.36 (0.04–3.07)	–
2015–2019	0.94 (0.57–1.54)	1.01 (0.60–1.71)	0.36 (0.03–4.04)	–
2020–2022	0.46 (0.17–1.29)	0.52 (0.18–1.47)	1.92 (0.25–15.00)	–
**% of 15–month periods with a cervical smear recorded**				
0–25%	1	1	1	1
26–50%	3.41 (1.68–6.93)	3.54 (1.68–7.48)	0.70 (0.19–2.61)	0.73 (0.19–2.77)
51–75%	4.65 (2.33–9.29)	4.71 (2.27–9.81)	0.48 (0.12–1.94)	0.77 (0.13–2.17)
>75%	7.72 (3.88–15.37)	8.12 (3.91–16.85)	0.19 (0.02–1.67)	0.18 (0.02–1.69)
**Previous cervical dysplasia diagnosis**				
No	–	–	1	–
Yes	–	–	3.24 (0.90–11.62)	–
**On antiretroviral therapy**				
No	1	1	^†^	–
Yes	1.22 (0.78–1.89)	1.16 (0.74–1.83)	–	–
**CD4 cell count at registration**				
Per 100 cells/μL increase	0.94 (0.90–0.99)	0.95 (0.90–1.00)	0.78 (0.61–1.01)	0.77 (0.60–0.98)

* Number of women included in crude analyses for variables without missing information.

^†^ All cervical cancers occurred in women on ART.

## DISCUSSION

Our study found a considerable burden of cervical dysplasia and cervical cancer among WWH in Switzerland and highlights persistent disparities in access to gynaecological care and cervical cancer screening in this population. Specifically, older women, those with a history of intravenous drug use, and women with lower education levels were less likely to undergo gynaecological visits. Furthermore, the likelihood of undergoing gynaecological visits decreased in the most recent calendar periods. When visiting a gynaecologist, women of Black or other ethnicities and those with low educational levels were less likely to have a cervical smear taken. However, compared to the 2001–2004 period, the likelihood of undergoing cervical smears during a gynaecological visit increased in subsequent years.

The main strength of this study lies in its extensive observation period. The SHCS is one of the longest‐running observational HIV cohorts worldwide, with participants followed up every 6 months. Standardized data collection forms and protocols ensure that high‐quality data on numerous biological and behavioural factors are captured at each study visit. Moreover, retention rates in the SHCS are high, reducing selection bias and enabling the assessment of trends over time and long‐term outcomes such as cervical dysplasia and cancer. Nevertheless, several limitations need to be considered. Self‐reported information on gynaecological visits and cervical smears can be subject to recall bias. However, the relatively short interval between consecutive SHCS study visits limits this concern. Still, overreporting might have occurred if participants mentioned the same gynaecological visits and cervical smears at more than one study visit. Social desirability bias might also have resulted in overreporting of gynaecological visits and cervical smears. Another limitation is that the gynaecological questionnaire did not capture detailed information on the reasons for the gynaecological visit or the indication and outcome of the cervical smear. Therefore, we could not clearly separate cervical cancer screening‐related visits from other gynaecological visits. The questionnaire also did not assess reasons why women did not undergo gynaecological visits. Furthermore, it is possible that some cervical dysplasias and cervical cancers diagnosed by external gynaecologists have not been captured in the SHCS. Therefore, our results may underestimate the cervical dysplasia and cancer burden among WWH in Switzerland. Lastly, while the SHCS includes approximately 70% of all people with HIV in Switzerland, health‐seeking behaviour may differ between SHCS participants and non‐participants.

Despite potential underreporting, we still found a considerable burden of cervical dysplasia and cancer among WWH in Switzerland. Our cervical cancer incidence estimate is similar to a study in the US [12] (26 per 100 000 person‐years), but substantially lower than what has been reported for WWH in Estonia [13] (106 per 100  000 person‐years) or Europe overall [14] (66 per 100 000 person‐years). These differences may be partly due to the variation in analytical approaches and study populations, but also differences in screening practices and uptake among WWH across these countries. While more regular cervical smears were associated with a higher likelihood of detecting cervical dysplasia in our analysis, they tended to be linked to a substantially lower cervical cancer risk. However, the association for cervical cancer was imprecise due to the small number of cancer diagnoses. We and others [15, 16, 17] found lower CD4 cell counts to be associated with an increased risk of cervical dysplasia and cervical cancer. Interestingly, in contrast to the results of a meta‐analysis [17], we did not find lower cervical dysplasia rates among women on ART, and all cervical cancer diagnoses occurred among women who had initiated ART. However, 96% of the women included in our study eventually initiated ART and, thus, our results regarding the impact of ART should be interpreted cautiously. Furthermore, our analysis confirmed cigarette smoking as an established risk factor for cervical dysplasia among WWH [18], possibly through the accumulation of smoke breakdown products in cervical mucus that can induce cell changes and impair local immune control [19]. Smoking cessation may help reduce cervical dysplasia and cancer risk among WWH [20].

Our findings reveal persistent disparities in the continuum of gynaecological care for WWH in Switzerland. A SHCS study published in 2006 showed that gynaecological care, including cervical cancer screening among WWH in Switzerland, was insufficient and needed to be improved [10]. The study identified various predictors for fewer gynaecological visits and cervical smears, including older age, non‐White ethnicity, lower education levels, and intravenous drug use. Almost two decades later, we still find that older women, those with a history of intravenous drug use, and women with lower education levels were less likely to undergo gynaecological visits. Notably, in our analysis, women of Black or other ethnicity were as likely as women of White ethnicity to report gynaecological visits. However, when visiting a gynaecologist, they were still less likely to have a cervical smear taken. Studies from other European countries, such as the UK [21] and Finland [22], identified various barriers to cervical cancer screening for non‐White women, including fear of racism, health care workers' negative attitudes, religious beliefs, lack of knowledge and awareness, language difficulties and embarrassment. To our knowledge, no such data on screening barriers for ethnic minorities are available for WWH or Switzerland specifically. The observed decline in gynaecological visits among older WWH may reflect limited awareness among health care providers and the women themselves about the continued importance of routine gynaecological surveillance in aging WWH. We also found a persistently low uptake of gynaecological visits among women with a history of intravenous drug use. This is in line with other studies documenting lower uptake of gynaecological appointments and cervical cancer screening among women with past or current substance use disorders [23, 24, 25]. Women with a history of drug use face many barriers to attending health care services and cervical cancer screening, including psychiatric comorbidities, other competing priorities, potential financial barriers, and stigma [26]. In general, more in‐depth quantitative and qualitative data are needed to better understand these persistent disparities in the continuum of gynaecological care for WWH in Switzerland. Between 2020 and 2022, women were substantially less likely to attend gynaecological visits, and tended to obtain cervical dysplasia diagnoses less frequently, likely reflecting the widespread strain on the health care system during the COVID‐19 pandemic. However, among those who did attend, the probability of having a cervical smear taken during a visit remained higher than in the 2001–2004 period, continuing a trend already observed in previous calendar years.

Switzerland lacks a systematic national or regional cervical cancer screening programme. While some women are under‐screened, others undergo cervical cancer screening more often than necessary [27]. Moreover, to date, there are no standardized screening guidelines for WWH, resulting in inconsistent practices across Switzerland. The SSGO suggests that women with immunosuppression, regardless of the cause, should be followed by experienced colposcopists without specifying a precise screening interval [9]; however, the practical implementation of this recommendation is left to the discretion of individual gynaecologists. To support more efficient and standardized care, there is a need for the development and dissemination of explicit cervical cancer screening guidelines for WWH in Switzerland and for raising awareness among both gynaecologists and infectious disease specialists. Integrating cervical cancer screening into HIV care through combined clinics has been shown to improve screening uptake by reducing the number of separate appointments and fostering closer collaborations between infectious disease specialists and gynaecologists [28, 29, 30]. Integration could also be facilitated by introducing hrHPV testing on self‐collected vaginal samples, a method which has the potential to increase screening uptake among under‐screened women [31, 32]. Offering a wider range of tailored screening strategies including clinic‐ or home‐based hrHPV self‐sampling might help reduce disparities across population groups [33]. Additionally, during health care disruptions such as the COVID‐19 pandemic, hrHPV self‐sampling could contribute to sustaining screening coverage [34]. However, data on the accuracy of hrHPV testing on self‐collected samples among WWH is still limited. Moreover, regular gynaecological visits remain essential to detect and treat precancerous anogenital lesions before cancer develops. WWH are at increased risk of various hrHPV‐associated cancers including vulvar, vaginal and anal cancer [35]. Since 2024, the International Anal Neoplasia Society recommends anal cancer screening for all WWH aged 45 years or above [36].

In conclusion, our study provides important insights into the gynaecological care of WWH in Switzerland and highlights substantial disparities in the uptake of gynaecological visits and cervical smears by age, education level, ethnicity and intravenous drug use status. Implementing more targeted and integrated cervical cancer screening and gynaecological care models may help reduce these disparities and improve cervical cancer prevention among WWH in Switzerland.

## AUTHOR CONTRIBUTIONS

KAP, KT and ER conceptualized the study, and all authors contributed to the design. KAP acquired the study funding. ER determined the methodology and performed the data analysis as well as the visualization of the data. Study findings were interpreted by KAP, ER and MW. MW, ER and KAP drafted the manuscript. All authors participated with their professional expertise in manuscript revision and approved the final version.

## FUNDING INFORMATION

This study has been financed within the framework of the Swiss HIV Cohort Study, supported by the Swiss National Science Foundation (SNSF Grant number #33FI‐0_229621, SHCS project number 900), and by the SHCS Research Foundation. The data are gathered by the Five Swiss University Hospitals, two Cantonal Hospitals, 15 affiliated hospitals and 36 private physicians. K Taghavi received funding from the Swiss National Science Foundation (210933).

## CONFLICT OF INTEREST STATEMENT

KAP's, AI's and AH's institutions have received travel grants and advisory fees from MSD, Gilead and ViiV Health care unrelated to this work. ON received expenses for a presentation on September 22, 2022 at the Satellite Symposium organized by ViiV Healthcare during the congress of the Swiss Society of Infectious Diseases and has no competing interests regarding this study. PET's institution reports grants, advisory fees, or educational fees from Gilead, ViiV, MSD, and Daiich‐iSankyo, outside the submitted work. All other authors report no conflicts of interest.

## Supporting information


**Table S1.** Crude and adjusted odds ratios (OR) for the association of different factors with the reporting of at least one gynaecological visit during a given 15‐month period.
**Table S2.** Crude and adjusted odds ratios (OR) for the association between different factors and undergoing a cervical smear (among those with a gynaecological visit) during a given 15‐month period.

## Data Availability

The data that support the findings of this study are available on request from the corresponding author. The data are not publicly available due to privacy or ethical restrictions.
