# Supplementary material for: Factors associated with uptake of gynaecological care and cervical cancer screening among women in the Swiss HIV Cohort Study
Source: HIV Med. 2025 Nov 11;26(12):1963–72. doi: 10.1111/hiv.70137 (PMC12666244; doi:10.1111/hiv.70137)
Supplement: Supplementary file 1 — Table S1. Crude and adjusted odds ratios (OR) for the association of different factors with the reporting of at least one gynaecological visit during a given 15‐month period. Table S2. Crude and adjusted odds ratios (OR) for the association between different factors and undergoing a cervical smear (among those with a gynaecological visit) during a given 15‐month period. [file HIV-26-1963-s001.docx]

**Supplementary Materials**

**Factors associated with uptake of gynaecological care and cervical cancer screening among women in the Swiss HIV Cohort Study**

**Supplementary Table 1:** Crude and adjusted odds ratios (OR) for the association of different factors with the reporting of at least one gynaecological visit during a given 15-month period.

**Supplementary Table 2:** Crude and adjusted odds ratios (OR) for the association between different factors and undergoing a cervical smear (among those with a gynaecological visit) during a given 15-month period.

**Supplementary Table 1:** Crude and adjusted odds ratios (OR) for the association of different factors with the reporting of at least one gynaecological visit during a given 15-month period.

|  | **Gynaecological visit** | | | | | |
| --- | --- | --- | --- | --- | --- | --- |
|  | **Crude OR (95% CI)** | | | **Adjusted OR (95% CI)*** | | |
| **Current age (years)** |  |  |  |  |  |  |
| 18-39 | 1 |  |  | 1 |  |  |
| 40-49 | 0.85 | (0.80 to | 0.90) | 0.92 | (0.86 to | 0.99) |
| 50-59 | 0.59 | (0.54 to | 0.63) | 0.69 | (0.63 to | 0.76) |
| ≥60 | 0.40 | (0.36 to | 0.45) | 0.45 | (0.39 to | 0.52) |
| **Ethnicity** |  |  |  |  |  |  |
| White | 1 |  |  | 1 |  |  |
| Black | 1.31 | (1.20 to | 1.43) | 1.01 | (0.91 to | 1.12) |
| Other | 1.34 | (1.18 to | 1.53) | 1.11 | (0.96 to | 1.28) |
| **Education** |  |  |  |  |  |  |
| Mandatory school or less | 1 |  |  | 1 |  |  |
| Apprenticeship | 1.08 | (0.98 to | 1.18) | 1.15 | (1.05 to | 1.26) |
| Bachelor or more | 1.32 | (1.18 to | 1.47) | 1.30 | (1.16 to | 1.46) |
| **History of intravenous drug use** |  |  |  |  |  |  |
| No | 1 |  |  | 1 |  |  |
| Yes | 0.59 | (0.53 to | 0.64) | 0.60 | (0.53 to | 0.67) |
| **Casual sexual partners in previous 6 months** |  |  |  |  |  |  |
| No | 1 |  |  | 1 |  |  |
| Yes | 1.29 | (1.17 to | 1.42) | 1.22 | (1.11 to | 1.34) |
| **Current CD4 cell count (cells/µL)** |  |  |  |  |  |  |
| <200 | 0.73 | (0.66 to | 0.82) | 0.62 | (0.55 to | 0.69) |
| 200-499 | 1.01 | (0.96 to | 1.07) | 0.91 | (0.85 to | 0.97) |
| ≥500 | 1 |  |  | 1 |  |  |
| **Calendar periods** |  |  |  |  |  |  |
| 2001-2004 | 1 |  |  | 1 |  |  |
| 2005-2009 | 1.07 | (0.99 to | 1.16) | 1.05 | (0.96 to | 1.15) |
| 2010-2014 | 0.94 | (0.86 to | 1.01) | 0.94 | (0.86 to | 1.03) |
| 2015-2019 | 0.81 | (0.75 to | 0.88) | 0.86 | (0.78 to | 0.94) |
| 2020-2022 | 0.62 | (0.56 to | 0.68) | 0.70 | (0.62 to | 0.78) |

* Adjusted for all other factors listed.

**Supplementary Table 2:** Crude and adjusted odds ratios (OR) for the association between different factors and undergoing a cervical smear (among those with a gynaecological visit) during a given 15-month period.

|  | **Cervical smear** | | | | | |
| --- | --- | --- | --- | --- | --- | --- |
|  | **Crude OR (95% CI)** | | | **Adjusted OR (95% CI)*** | | |
| **Current age (years)** |  |  |  |  |  |  |
| 18-39 | 1 |  |  | 1 |  |  |
| 40-49 | 0.93 | (0.88 to | 0.99) | 1.17 | (1.04 to | 1.31) |
| 50-59 | 0.69 | (0.63 to | 0.74) | 1.21 | (1.03 to | 1.43) |
| ≥60 | 0.47 | (0.42 to | 0.53) | 0.85 | (0.67 to | 1.07) |
| **Ethnicity** |  |  |  |  |  |  |
| White | 1 |  |  | 1 |  |  |
| Black | 1.15 | (1.06 to | 1.26) | 0.78 | (0.67 to | 0.90) |
| Other | 1.19 | (1.05 to | 1.35) | 0.85 | (0.69 to | 1.05) |
| **Education** |  |  |  |  |  |  |
| Mandatory school or less | 1 |  |  | 1 |  |  |
| Apprenticeship | 1.15 | (1.06 to | 1.26) | 1.31 | (1.14 to | 1.51) |
| Bachelor or more | 1.32 | (1.19 to | 1.47) | 1.20 | (1.02 to | 1.42) |
| **History of intravenous drug use** |  |  |  |  |  |  |
| No | 1 |  |  | 1 |  |  |
| Yes | 0.67 | (0.61 to | 0.73) | 1.09 | (0.92 to | 1.30) |
| **Casual sexual partners in previous 6 months** |  |  |  |  |  |  |
| No | 1 |  |  | 1 |  |  |
| Yes | 1.28 | (1.16 to | 1.40) | 1.14 | (0.96 to | 1.35) |
| **Current CD4 cell count (cells/µL)** |  |  |  |  |  |  |
| <200 | 0.73 | (0.66 to | 0.82) | 0.89 | (0.71 to | 1.11) |
| 200-499 | 0.99 | (0.93 to | 1.04) | 0.96 | (0.86 to | 1.07) |
| ≥500 | 1 |  |  | 1 |  |  |
| **Calendar periods** |  |  |  |  |  |  |
| 2001-2004 | 1 |  |  | 1 |  |  |
| 2005-2009 | 1.19 | (1.10 to | 1.28) | 1.52 | (1.31 to | 1.78) |
| 2010-2014 | 1.01 | (0.94 to | 1.09) | 1.21 | (1.04 to | 1.42) |
| 2015-2019 | 0.91 | (0.84 to | 0.99) | 1.24 | (1.05 to | 1.47) |
| 2020-2022 | 0.72 | (0.66 to | 0.79) | 1.26 | (1.03 to | 1.54) |

* Adjusted for all other factors listed.
